# Overgeneralisation Effect in Trait Inferencing About a Child with Craniofacial Microsomia by Medical Students and Child’s Relatives

**DOI:** 10.34763/jmotherandchild.20202403.2025.d-20-00010

**Published:** 2021-01-29

**Authors:** Katarzyna A. Milska, Arkadiusz Mański, Jolanta Wierzba

**Affiliations:** 1 Department of Quality of Life Research, Faculty of Health Sciences with Institute of Maritime and Tropical Medicine, Medical University of Gdansk, Gdańsk, Poland; 2 Psychological Counselling Centre for Rare Genetic Diseases, Institute of Psychology, University of Gdansk, Gdańsk, Poland; 3 Kashubian Institute of Gdansk, Gdańsk, Poland; 4 Department of Pediatric and Internal Medicine Nursing, Medical University of Gdansk, Gdańsk, Poland

**Keywords:** craniofacial microsomia, face processing, overgeneralisation effect

## Abstract

**Background:**

The study uses the *Overgeneralisation Effect Scale* (*OES*) developed by K. Milska and A. Mański to estimate the overgeneralisation effect in trait inferencing about children with craniofacial anomalies, which involved university students (future health professionals) and relatives of children with craniofacial microsomia (*CFM*). The practical purpose of the study was to provide evidence supporting the benefits of using the *OES* to improve the outcomes of child rehabilitation.

**Methods:**

The *OES* (Polish: *Skala Efektu Nadgeneralizacji*) was administered to a group of 843 university students of medical/caring professions and 26 parents/guardians of children with craniofacial anomalies. The responses of 757 subjects were included in the analysis.

**Results:**

Different trait profiles of a child with *CFM* were obtained. The carer appraisal of their child tended to be very positive across all items. The student appraisals were definitely less positive and more varied. A range of factors which may affect trait impression leading to overgeneralisation in trait inferencing about a child with *CFM* have been identified, including familiarity with the child, craniofacial anomaly suggestive of more severe disability, emotional expression and the relationship to the child.

**Conclusion:**

The behaviour of the carers and professionals towards children with *CFM* undergoing diagnostic assessment, treatment and rehabilitation is determined by how each of them perceives the child. The presence of anomaly increases the likelihood of overgeneralisation effect both in carers and professionals. The *OES* may be one of the instruments to detect/measure these differences to improve the outcomes of child rehabilitation

## Introduction

An exceptionally complex object which constantly changes in response to the external stimuli, whilst maintaining fixed elements to ensure its physical configuration and physiognomy, a human face is a significant contributor to human interaction. Both invariant and changeable aspects of face are perceived by others who can ascribe different meanings to them.^1, 2, 3^

Before a set of facial cues becomes a social message playing an important role in interaction, these have to undergo complex processing algorithms. Currently, there are three main research and applied concepts to explain these algorithms by means of e.g. the underlying neural processes: the Bruce and Young model of face recognition, the Haxby model and the concept of configural processing by Maurer, Le Grand and Mondloch.^4^

The Bruce and Young’s model indicates that face processing may lead to face recognition and person identification. The face recognition units and person identity nodes are markedly different which becomes particularly obvious during the exposure to various input structural codes (e.g. physical features, voice and speech, and clothing items). It should be noted that even if a person is unknown, some person identity nodes may still be activated; for instance, it is still possible to determine their sex, age, health condition, ethnicity or intelligence. However, the situation is completely different when a person is familiar, as only person identity nodes are activated then at all stages of face processing.^5, 6^

On the other hand, the Haxby model^7^ emphasises neural systems of face perception. Face processing involves a number of distributed cerebral structures, which enable person identification. This model differentiates between the invariant and changeable aspects of faces. The invariant aspects of faces are of genetic origin, and as such are predetermined by widely understood inheritance. In an interaction, they may serve as cues to determine personal details, certain biographic aspects or unique identifiers. These aspects are processed primarily by the lateral fusiform gyrus and anterior temporal regions. The neural system responsible for processing the changeable aspects of faces, such as eye gaze, lip movement, spatially directed attention, prelexical speech and emotional expression, is more complex and includes such key structures as the superior temporal sulcus, the intraparietal sulcus, the auditory cortex, the amygdala, the insula and the limbic system.^7, 8^

The concept of configural processing particularly accentuates the relations between individual parts of the face. The approach postulates three types of configural processing. Initially, the stimulus is assigned to the appropriate first-order relations. Then, the key elements of the perceived stimulus are processed holistically (as gestalt), which forms a single face image. Finally, the second-order relations are identified which require information on relative position, size and colour of face elements. Undoubtedly, this approach assumes the existence of a prototypical face and the possibility to create novel, unique and cognitive representations for each face.^9, 10^ In the light of the presented face processing models, a question may arise regarding factors contributing to the complexity of this process. Our current understanding links it to the evolutionary changes to the human brain due to interaction with other people, especially the trend to form larger communities.^11^ In larger communities, mere face recognition may have proved insufficient to serve adaptive action. The face is no longer a purely physical object; at times it is affected by emotions, but it has become the source of information that extends far beyond its directly available physical aspect. As pointed out by Gibson,^12^ other people’s faces provide a set of adaptive cues that guide social perceptions and enable appropriate response in an interaction. In most cases, these cues are processed in an uninterrupted manner. However, Zebrowitz and Montepare^13^ notice that there are situations, in which perceiver’s attunement to stimulus information may be needed, resulting in overgeneralised perceptions. Two of them have been explored in particular, i.e. the anomalous face and the babyface overgeneralisation effect.^14^

Facial attractiveness is one of the most well-researched aspects of cognitive psychology. Better looking people are undoubtedly perceived more positively across all dimensions. They may, for instance, receive more favourable reactions from the community.^15, 16^ On the other hand it should be mentioned that faces perceived as unattractive resemble those with genetic anomalies which often results in such trait inferences as lower emotional warmth, physical weakness or lower competence.^17^

Another phenomenon, which may give rise to the overgeneralisation effect, is the so-called ‘babyface’. A mere encounter of a babyface triggers positive responses.^18, 19^ A babyface is described as rounder, with a narrow chin, higher forehead, smaller nose and plump lips. Furthermore, the babyface phenotype entails larger eyes and high raised eyebrows. Such a constellation of features at least partially noticeable on an adult face may trigger the impression that such a person is warm, caring and honest, yet also less intellectually and socially competent.^14, 20^

As shown above, facial cues may affect one’s relations with others in a complex way. Face processing can not only lead to recognising the stimulus as a human face but also to trait inferencing, even if the beliefs are unfounded. The ultimate result is a set of features which enable one to adapt to the interaction with someone they have appraised.

The overgeneralisation effect may have detrimental consequences for the social functioning of individuals with facial anomalies. A comprehensive review of these consequences may be found in the article by Riklin, Andover and Annunziato,^21^ who focused on the psychosocial functioning of adolescents with craniofacial conditions (CFCs). In their proposed psychosocial model of social dysfunction in adolescents with CFCs, they provide a clear hypothesised pathway to explain possible social maladjustment in this group, identifying possible perpetuating factors such as unattractive facial appearance, social stigma as well as lack of social awareness and understanding among their peers. They ultimately lead to loneliness, low self-confidence and lack of social support.^21, 22^

However, not all individuals with facial anomalies experience the negative consequences of overgeneralisation. Numerous studies show that where facial appearance changes as a result of cancer treatment or blindness, the social response may be exceptionally positive. This may, at least partly, be explained by better awareness of cancer treatments and situation of such individuals.^23^ A few studies have shown that facial anomalies in children do not necessarily lead to negative overgeneralisation. For individuals with numerous yet minor anomalies, these ‘defects’ may even go disregarded/ unnoticed. This phenomenon was demonstrated to occur in mothers and teachers directly involved with children with moderate learning disability presenting numerous facial anomalies.^10, 24^ It shows that the overgeneralisation effect is a complex phenomenon underscored by multiple factors and its mechanisms have not as yet been explained.

The aim of the study was to assess the size of the overgeneralisation effect in the perception of a child with CFM by medical university students and people who are close to the child.

The practical aim was to indicate the legitimacy of considering the role of the overgeneralisation effect at the beginning of the treatment and the rehabilitation process of a child with facial and body deformities to improve the effectiveness of treatment and rehabilitation. The size of the discrepancies shows how the rehabilitation process will look like in the interpersonal dimension (climate, emotions, interpersonal communication with relatives and the child). Reducing the discrepancies in the images shaped by specialists and caregivers of children with CFM can help improve the effectiveness of the treatment and the rehabilitation process.

## Methods

### Participants

The study group consisted of 843 university students and 26 parents/carers of children with craniofacial microsomia (*CFM*). The uniqueness of the face of children with *CFM* lies, among others, in the fact that deformities cover a limited part of the face (in some cases it is possible to even meet the underdevelopment of half of the face with the properly built other part). The enrolled students were future healthcare professionals, from the medicine, dentistry, nursing and health psychology at the Medical University of Gdańsk. The selection of students was random. The only selection criterion was the field of study. The study was conducted among students due to the educational purpose of the study. The students were supposed to be the future healthcare professionals who had no work experience at the time of the study. Both students of medicine and dentistry were doctors to be, while only the second of the groups from all of students mentioned during their fourth year of study had the opportunity to obtain very specialised knowledge in the field of face and head anatomy, which might have influence on the effectiveness of defect detection.

Parents/carers were surveyed during the conference for parents/carers of children with *CFM* held at Centre for Craniofacial Anomalies and Oral and Maxillofacial Surgery in Olsztyn and during individual meetings with parents/carers who are attending the Psychological Counselling Centre for Rare Genetic Diseases at the Institute of Psychology, University of Gdansk.

The participation in the study was voluntary, and the study was made with the consent of the participants. This scientific project was approved by the Independent Bioethical Committee for Scientific Research at the Medical University of Gdańsk (NKBBN/48/2017, NKBBN/178/2018) and the management of the Provincial Specialist Children’s Hospital named after

**Table 1:** Characteristics of enrolled students and parents/carers of children with CFM.

University Department/ Programme	*n* before analysis	*n* rejected in analysis	Group size (*n*)	Group size (%)
Medicine	568	86	482	65.22%
Dentistry	130	10	120	16.24%
Nursing	100	6	94	12.72%
Health psychology	45	2	43	5.82%
Total	843	104	**739**	**100%**
Parents/Carers of children with CFM	26	8	**18**	**100%**

Prof. Stanisław Popowski in Olsztyn. The participants who did not respond to at least one item (a pair of adjectives) in *OES* or did not provide their informed consent to participate in the study were excluded from analysis.

### Procedures

Having provided their written consent to participate in the study, the participants were requested to complete the Milska and Mański’s *Overgeneralisation Effect Scale* (*OES*) appraising a child with *CFM*. The students were provided a visual stimulus of a child with *CFM* in photographs from the book *Syndromes of the Head and Neck*,^25^ whereas parents/carers assessed their child with CFM. The assessed children did not differ in ethnicity. The version of *OES* by Milska and Mański consists of 20 bipolar 7-point scales. The child is assessed in terms of the following dimensions: *Dexterity(D), Personality Traits (PT), Values (V), Appearance (A)* and *Overall score* (sum of the scores of all scales). Each of the 4 factors includes 5 bipolar scales.

The reliability of *OES* determined using Cronbach’s a is 0.80. The total score ranges between 20 and 140, with the scores of 5 to 35 on individual factors. The *OES* was used to find out how a person is perceived in different dimensions. Despite the static photo, when the person appears, the other one concludes about his features without even knowing him, which is the essence of the phenomenon of overgeneralisation.

## Results

**Figure 1:**
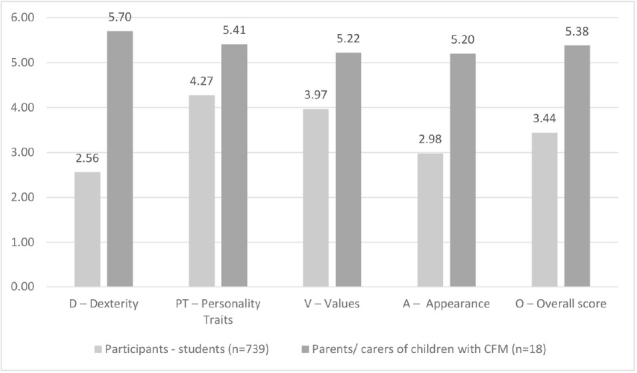
Mean values (*M*) for factors and overall score obtained by the students and parents/carers of children with CFM.

Student responses provided a relatively variable child trait profile (Figure 1). Whereas the *Dexterity* and *Appearance* scores were lower than the *Overall score* (*M* = 3.44), the *Personality Traits* and *Values* scores were higher than the *Overall* score. Interestingly, the students appraised the *Personality Traits* of a presented child the highest (*M* = 4.27), simultaneously providing lowest rates to the child’s *Dexterity* (*M* = 2.56). Except for traits directly associated with the child’s appearance, the remaining trait inferencing had to be indirect, with significant pieces of information missing. Nevertheless, it seems important that it is the child’s appearance is the primary source for the indirect trait inferencing, which the remaining sources can base upon in such a design.

Parent/carer responses provided a highly invariable trait profile of a child (Figure 1). Whereas the *Dexterity* and *Personality Trait* scores were just slightly higher than the *Overall* scores (*M* = 5.38), the *Appearance* and *Values* scores were lower than the *Overall* scores. Interestingly, parents/carers rated their child’s *Dexterity* the highest (*M* = 5.70) but their *Appearance* the lowest (*M* = 5.20). It should be mentioned that such scores indicate a very positive and fairly invariable appraisal of a child. Unlike future healthcare professionals, parents do not rely on indirect trait inferencing. Their perception of the child is multi-contextual and by providing nurturing, they contribute to developing their child’s image on a daily basis.

**Figure 2:**
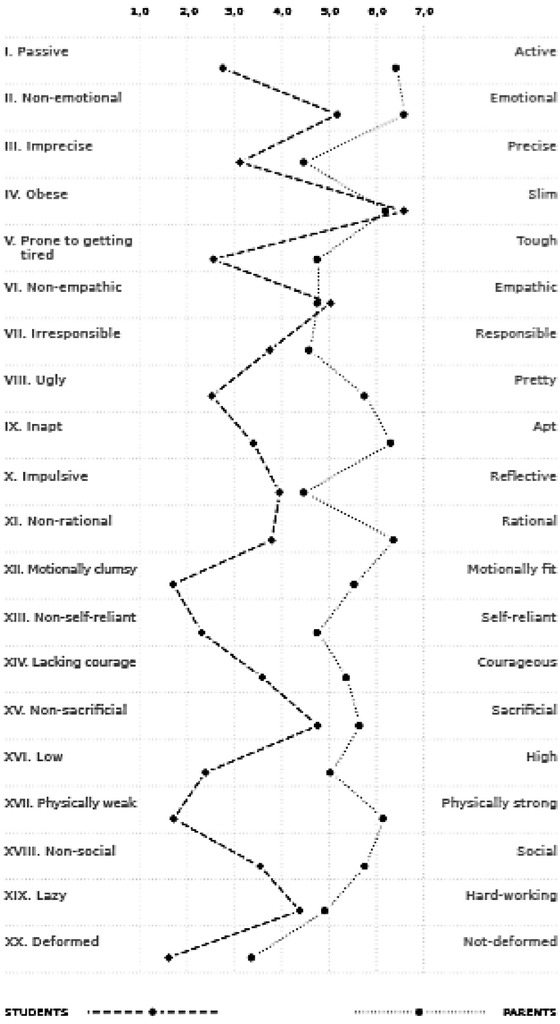
Mean values (*M*) of individual OES item scores in students (*n*=739) and parents/carers of children with CFM (*n*=18).

Comparing the individual *OES* items during the analysis led to particularly intriguing findings (Figure 2). The results were only similar on 6 of 20 items (scales: IV, VI, VII, X, XV and XIX). There was a level of agreement between future health care professionals and parents/carers on a positive appraisal of such traits as slim body, empathy, responsibility, reflectiveness, self-denial and diligence in a child with *CFM*. On the remaining 14 items, however, the perception of a child seems to diverge significantly between parents/ carers and students and polarise (positive – negative). At this point, extreme scores need to be mentioned. Students provided particularly low rating on scales XII, XVII and XX, thereby inferring that the child in the photograph is clumsy, physically weak and very deformed. They also state that the presented child is very slim (*M* = 6.59), which is the only extreme positive score in this group of respondents. On the other hand, the parental appraisal is strongly shifted towards the positive. Parents/carers provided extremely high scores on 6 out of 20 items (I, II, IV, IX, XI and XII), thereby indicating that their child is very active, emotional, quite slim, gifted, smart and exceptionally physically fit. The largest difference between the student and parent/carer scores was observed on item XII.

## Discussion

From a psychological point of view, facial and thoracic anomalies may be considered in connection with stigma. As pointed by Goffman,^26^ in certain circumstances minor and major deformities may become a stigma, a mark of social disgrace. This paper provides a comparison and analysis of responses on trait inferencing about a child with *CFM* by their parents and future healthcare professionals. Intriguingly, the clash of those discrepant views often occurs in a real-life setting between parents and healthcare professionals in their working relationship e.g. during rehabilitation, where a child with *CFM* is a subject of the interaction. Even the preliminary analysis of trait profiles obtained using *OES* indicates that they differ significantly. When images of the child are established, one of the goals of rehabilitation may be to reduce this interpersonal discrepancy. The effectiveness of rehabilitation depends on how parents and experts manage to bring these images closer (e.g. through the process of mutual negotiations) – increasing their compatibility may contribute to improving the effectiveness of rehabilitation and improving the child’s social functioning.

As Zebrowitz indicates,^3^ the physical appearance is one of the first aspects that people base their trait impressions and inferences on, which poses the risk for significantly distorted and superficial appraisals. On the other hand, despite strong evidence for the effect of some facial cues on perceiver’s trait impressions, their contribution to overgeneralisation effect remains unconscious. Many authors point out that overgeneralisation effect can be evoked in response to such cues as a babyface or strong childlike features on an adult’s face, familiar face, fitness traits and emotional expression.^27, 28, 29, 30^ The diversity of our results can be at least partly explained by this set of cues. Our participants were to appraise a child with *CFM*. The difference in the mode of exposure between students and parents/carers may have significantly affected their perception. The students saw a child in a photograph for a short while, whereas the parents/carers spend time with their child almost every day. Willis and Todorov^31^ notice that too short exposure to different aspects of appearance is a possible source of interference. Unfortunately, it seems to be enough for the interference to significantly affect a number of key decisions regarding trait inferencing.^32, 33, 34^ The overgeneralisation effect may also occur when one is exposed to an unfamiliar object or an object presented in an unattractive way.^35^ For the students in our study, the child with *CFM* shown in the picture was not only unknown but also unattractive – unlike to the parents/carers who perceived their child as familiar and who contributed to their image (e.g. clothing and background). Another cue could be relative position of facial elements to ascertain the emotions expressed. The photograph only captures a small part of those relations, which is why the students did not have the opportunity to perceive the dynamic changes on the face that could have improved their judgement accuracy so as to the type and intensity of emotions expressed. Importantly, facial dysmorphia further impairs the recognition and interpretation or emotional expression. With parents, though, their appraisal may be more accurate due to personal long-term experience with their children. It should be mentioned that the obtained profiles may have adaptive meaning. The perception of, and hence trait profile provided by a future healthcare professional may be affected by their role and the working relationship with the child with facial and thoracic anomalies during their treatment and rehabilitation.

For the close relatives, though, this role is completely different, hence the trait profile with a clearly positive and internally consistent score distribution offers an opportunity to encourage behaviours beyond the standard treatment and rehabilitation. Thinking of adaptation, it should be noted that the trait profile provided by parents/carers is particularly positive. As shown by Mański,^2^ mothers of children with numerous facial anomalies tend not to notice or disregard these anomalous features. Therefore, their further appraisals and inferences may be determined by the facial stimulus after a ‘deformity retouch’. It may be, e.g. a face of the child the mother dreamt and created an internal image of before the actual child with genetic defect was born. For the students, on the other hand, the appraisal of the child in the photograph is likely the result of comparison with other children they encountered during their training or in private life. This variety of sources could translate into internally inconsistent score profile in students.

As indicated by Kowalik,^36^ the rehabilitation process entails an interaction between e.g. patients, their relatives, doctors, physiotherapists, nurses or psychologists. Each participant brings knowledge of themselves, of the situation and of themselves in the situation into the interaction. Where there are so many, often clashing, perspectives, the atmosphere may be created which is conducive or detrimental to treatment. Any discrepancies may be minimised through negotiations, which also affect the overgeneralised image of a patient.^37^ The trait profiles of a child with *CFM*, obtained from students and parents/carers, indicate significant discrepancies. However, this situation is likely typical for all first-time encounters of a child with *CFM* at the beginning of their diagnostic assessment, treatment and rehabilitation.

The professional appraisal of a child with *CFM* may be more inconsistent. The experience shows, however, that the perception of these children mainly by their mothers is usually very positive, and the individual trait profiles closely resemble one another. To sum up, our study supports the need to identify the factors that can facilitate reducing the perceptual discrepancies. Last but not least, it should be emphasised in professional training that health care professionals working with patients with facial anomalies are more likely to use a cognitive mechanism of overgeneralisation. selected sample of caregivers’ group may not reflect the general population (sample bias). Despite the small group of participants, the researchers noticed certain patterns in the examined group. Despite the promising results of our study, further studies are necessary.

## Key points

- The rehabilitation process entails an interaction between e.g. patients, their relatives and professionals (doctors, physiotherapists, nurses or psychologists).
- The trait profiles of a child with *CFM*, obtained from students and parents/carers, indicate significant discrepancies.
- The perception of children with *CFM* mainly by their mothers is usually very positive and the individual trait profiles closely resemble one another.
- The professional appraisal of a child with *CFM* is more inconsistent.
- *The Overgeneralisation Effect Scale* (*OES*) may be one of the instruments to detect/measure differences in the perception of a child with *CFM* between the relatives and the professionals.
- An attempt to reduce the differences in the perception of a child with *CFM* between the relatives and the professionals involved in their diagnostic assessment and treatment is justified to improve the outcomes of child rehabilitation.
